# CapZyme-Seq: A 5′-RNA-Seq Method for Differential Detection and Quantitation of NAD-Capped and Uncapped 5′-Triphosphate RNA

**DOI:** 10.1016/j.xpro.2019.100002

**Published:** 2020-06-03

**Authors:** Irina O. Vvedenskaya, Bryce E. Nickels

**Affiliations:** 1 Department of Genetics and Waksman Institute, Rutgers University, Piscataway, NJ 08854, USA

## Abstract

Nucleoside-containing metabolites such as the oxidized and reduced forms of nicotinamide adenine dinucleotide (NAD^+^ and NADH), 3′-desphospho-coenzyme A (dpCoA), and flavin adenine dinucleotide (FAD) can be incorporated as RNA 5′ end caps by serving as non-canonical initiating nucleotides (NCINs) for transcription initiation by RNA polymerase. We recently reported “CapZyme-seq,” a 5′-RNA-seq method that enables the differential detection and quantitation of relative yields of NCIN-capped RNA and uncapped 5′-triphosphate RNA. Here we provide the protocol for constructing cDNA libraries for CapZyme-seq.

For complete information on the generation and use of this protocol, please refer to Vvedenskaya et al. (2018a).

## Highlights

• Protocol for high-throughput detection and quantitation of NAD^+^-capped RNA
• Method enables analysis of capping for RNA generated both *in vitro* and *in vitro*
• Analysis of relative efficiency of NAD^+^- versus NTP-mediated transcription initiation

## Graphical Abstract

**Figure:**
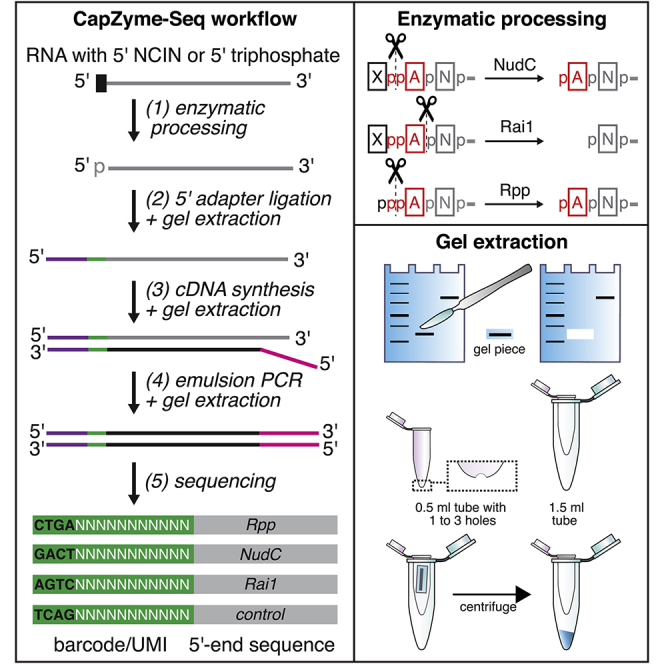

## Before You Begin

**CRITICAL:** Use nuclease-free H_2_O at all steps whenever H_2_O is needed.

**CRITICAL:** Perform all steps in a nuclease-free environment.

### Source of Input RNA

Libraries can be prepared using RNA generated *in vitro* (part A) or *in vivo* (part B) as input.

### Isolation of RNA Products Generated *In Vitro*

For CapZyme-seq analysis of RNA products generated *in vitro*, the experimenter controls the identity of the NCIN added to transcription assays, and thus controls the identity of the NCIN-capped RNA products used as input. Transcription assays are performed in the presence and absence of an NCIN; results obtained for assays performed in the absence of an NCIN are used as a negative control, or background correction (Vvedenskaya et al., 2018a). We recommend performing transcription assays in triplicate. If necessary, *E. coli* RNA polymerase can be replaced by another RNAP (e.g., T7 RNAP).

#### Transcription Assay

**TIMING: ∼3 h**

1. Mix reagents for *in vitro* transcription assays as indicated below.

**Table:** 

Reagents	Volume, μL
100 nM template DNA	10
500 nM RNAP holoenzyme	10
500 mM Tris-HCl pH 8.0	8
1M MgCl_2_	1
0.1 mg/mL BSA	10
1M KCl	10
25% glycerol	20
100 mM DTT	10
RNaseOUT, 40 U/μL	1
**Total:**	80

2. Incubate the mixture at 37°C for 10 min to form RNAP-promoter open complexes.
3. Prepare NTP mixtures with or without an NCIN (e.g., NAD^+^) as indicated below and incubate at 37°C for 10 min.

**Table:** 

Reagents	Assay with NTPs and NAD^+^ Volume, μL	Assay with NTPs Only Volume, μL
ATP, 5 mM	2	2
CTP, 5 mM	2	2
GTP, 5 mM	2	2
UTP, 5 mM	2	2
NAD^+^, 100 mM	2	-
Heparin, 10 μg/μL	1	1
500 mM Tris-HCl pH 8.0	2	2
H_2_O	7	9
**Total:**	20	20

4. Initiate a single round of transcription by adding an NTP mixture (prepared in 3) to the solution containing RNAP-promoter open complexes (prepared in 2). Incubate at 37°C for 15 min and stop the reactions by adding 0.5 M EDTA (pH 8) to a final concentration of 50 mM.
5. Add 10 μL of 3 M NaOAc, mix by vortexing for 30 s, add 330 μL of 100% EtOH, and precipitate nucleic acids at −80°C for 16 h.
6. Recover the nucleic acids by centrifugation for 30 min at 21,000 x *g* at 4°C, wash pellets 3 times with cold 80% EtOH, resuspend in 30 μL of nuclease-free water, and add 30 μL of 2x RNA loading dye.

#### Isolation of RNA Products (Figure 1)

**TIMING: ∼4 h**

1. Load sample (from step 6 above) on a 10% TBE-urea slab gel along with a ssRNA size ladder. Run gel in 1x TBE buffer until bromophenol blue dye front reaches ∼3/4 of the length of the gel. Samples for distinct transcription reactions should be run using separate gels.
2. Incubate gels with SYBR Gold nucleic acid gel stain and visualize bands with UV transillumination. Excise RNA products of the expected size from the gel using a scalpel. Gels for distinct transcription reactions should be stained in separate containers.
3. Assemble two tubes to crush the gel pieces. Pierce one to three holes in the bottom of a 0.5 mL low binding microfuge tube with a 23-gauge needle. Place this tube into a 1.7 mL low binding microfuge tube. Place gel slices into the upper tube and centrifuge the tube-in-tube assembly at 14000 *x g* for 2 min or until all gel fragments have passed through the holes in the upper 0.5 mL tube into the lower 1.7 mL tube.
4. Add 400 μL of gel elution buffer (0.3 M NaCl, 10 mM Tris-HCl pH 8, 0.1 mM EDTA) to the crushed gel and incubate the slurry at 70°C for 10 min. Mix by vortexing for 30 s, transfer the slurry to a Spin-X tube filter, centrifuge at 14,000 *x g* for 1 min, and transfer the eluate into a 1.7 mL tube.
5. Remove the crushed gel from the Spin-X tube, mix with 400 μL of gel elution buffer, and repeat steps described in step 4 above.
6. Combine the eluates from steps 4 and 5, add 80 μL of 3M NaOAc and 8.8 μL of glycogen (10 mg/mL), mix by vortexing for 30 s, add 650 μL of isopropanol, mix by vortexing for 30 s, and precipitate RNA at −80°C for 16 h.

**PAUSE POINT:** RNA can be stored at −80°C for ∼6 months.

7. Centrifuge tubes at 4°C for 30 min at 21,000 x *g* to pellet RNA. Wash pellets 3 times with 1 mL of 80% ice-cold EtOH.
8. Allow the pellet to air-dry for ∼5 min. Resuspend RNA in 10 μL of nuclease-free water. Pipette the suspension and incubate on ice for at least 20 min to fully resuspend the RNA.

***Note:*** The amount of RNA generated in four 100 μL reactions is sufficient for use in two separate enzymatic treatments (Rpp and NudC or Rai1) and two distinct control reactions (mock Rpp treatment and mock NudC or mock Rai1 treatment; Vvedenskaya et al., 2018a).

**Figure 1:**
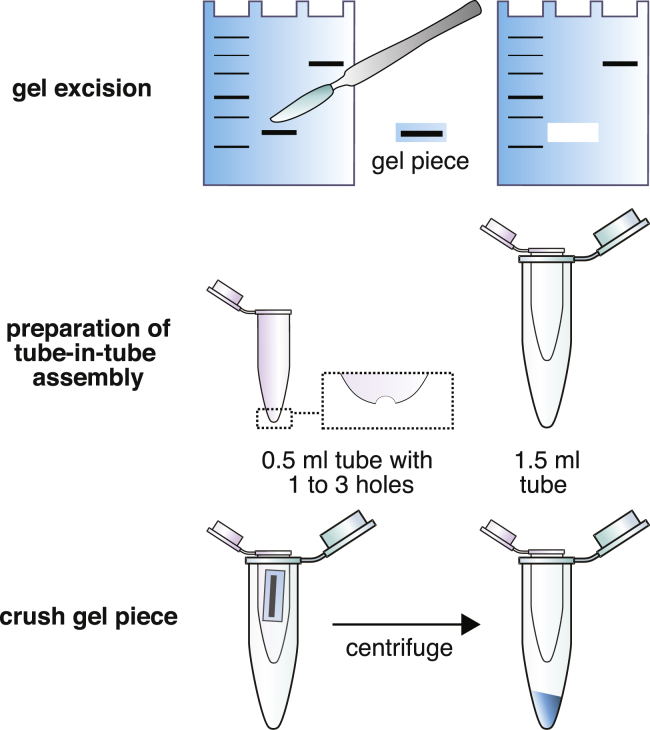
Isolation of Nucleic Acids by Gel Extraction

### Isolation of RNA Products Generated *In Vivo* (in *E. coli*)

We recommend use of TRI Reagent solution to isolate RNA from *E. coli* cultures as described in Vvedenskaya et al. (2018b).

#### Cell Culture and Harvest

**TIMING: ∼2 days**

1. Place 5 mL of LB media in a 15 mL culture tube and inoculate with *E. coli*. Grow culture overnight at 37°C on a roller drum set to 210 RPM.
2. Add 0.5 mL of the overnight culture to 50 mL of LB media in a 250 mL DeLong flask. Grow culture at 37°C on a platform shaker at 210 RPM to mid-exponential phase (OD600 ∼0.5).
3. Harvest 2 mL of cell suspension into 2-mL tubes and collect cells by centrifugation for 1 min at 21,000 x *g* at 25°C. Remove supernatant and freeze cell pellets rapidly on dry ice. Store cell pellets at −80°C.

**PAUSE POINT:** Cell pellets can be stored at −80°C for ∼6 months.

***Note:*** Use one cell pellet for RNA isolation, store the remaining pellets at −80°C as a back up.

#### RNA Isolation

**TIMING: ∼3 h**

1. Resuspend cell pellet in 600 μL of TRI Reagent solution. Incubate at 70°C for 10 min and centrifuge for 10 min at 21,000 x *g* at 4°C to remove insoluble material. Transfer supernatant to a fresh tube.
2. Add 100% EtOH to a final concentration of 60.5% and divide the mixture equally between two Direct-zol spin columns (Zymo Research). Perform DNase I treatment on-column according to the manufacturer’s recommendations.
3. Elute RNA from the column using 30 μL of nuclease-free water heated to 70°C. Repeat the elution two more times. Pool the eluates.
4. Add 10 μL of TURBO DNase 10x buffer and 1 μL of TURBO DNase to the recovered RNA. Incubate the mixture at 37°C for 30 min to remove residual DNA.
5. Purify RNA using acid phenol:chloroform pH 4.5.
  a. Add nuclease-free H_2_O to TURBO DNase-treated RNA to a total volume of 200 μL, mix with 200 μL of acid phenol/chloroform pH 4.5. Mix by vortexing for 10 s.
  b. Centrifuge for 30 s at 21,000 x *g* at 25°C and recover the upper aqueous phase containing RNA.
  c. Add 200 μL of chloroform to remove residual phenol, mix by vortexing for 10 s. Centrifuge for 30 s at 21,000 x *g* at 25°C and remove the majority of the lower organic phase. Centrifuge for 1 min at 21,000 x *g* at 25°C and remove the residual organic phase.
  d. Add 20 μL of 3M NaOAc and 2.2 μL of glycogen (10 mg/mL), mix by vortexing for 30 s, add 670 μL of 100% EtOH, mix by vortexing for 30 s, and precipitate RNA at −80°C for 16 h.
    **PAUSE POINT:** RNA can be stored at −80°C for ∼6 months.
  e. Centrifuge at 4°C for 30 min at 21,000 x *g* to pellet RNA. Wash pellets 3 times with 1 mL of 80% ice-cold EtOH.
  f. Air-dry the pellet for ∼5 min. Resuspend RNA by addition of 50 μL of nuclease-free water. Pipette the suspension and incubate on ice for at least 20 min to permit complete resuspension of the RNA. Measure the concentration using a NanoDrop.
    ***Note:*** The typical yield of RNA isolated from 2 mL of an *E. coli* cell suspension is ∼60 μg.

#### rRNA Depletion

**TIMING: ∼3 h**

1. Use MICROBExpress Kit to deplete rRNAs from 9 μg of recovered RNA according to the manufacturer’s recommendations.
2. Measure the volume of rRNA-depleted RNA separated from beads. Add 10% volume of 3M NaOAc and 1% volume of glycogen (10 mg/mL), mix by vortexing for 30 s, add 3 volumes of 100% EtOH, and precipitate RNAs at −80°C for 16 h.

**PAUSE POINT:** RNA can be stored at −80°C for ∼6 months.

3. Recover RNA as in (**B**, RNA isolation, step 5e-f). Resuspend RNA in 30 μL of nuclease-free water. Measure the concentration with a NanoDrop.

***Note:*** We recommend using 2 μg of rRNA-depleted RNAs as a minimum starting amount for each enzymatic processing reaction described below.

#### CIP Treatment of RNA Prior to NudC or Rai1 Treatments

**CRITICAL:** For analysis of NCIN-capped RNA, we recommend treating RNA with Calf Intestinal Phosphatase (CIP) to remove 5′ end phosphates prior to performing the NudC or Rai1 treatments described below.

1. Assemble the reaction as indicated below.

**Table:** 

Reagents	Volume (μL)
rRNA-depleted RNA, 2 μg	10
10x CutSmart Buffer	3
RNaseOUT, 40U/μL	1
CIP, 10U/μL	0.2
H_2_O	15.8
**Total:**	30

2. Incubate the reaction at 37°C for 1 h.
3. Add nuclease-free H_2_O to 200 μL, mix with 200 μL of acid phenol/chloroform (pH 4.5), and purify RNA using the procedure described in (**B**, RNA isolation, step 5a-f).
4. Resuspend the RNA pellet in 10 μL of nuclease-free water. Pipette the suspension and incubate on ice for at least 20 min to ensure the complete resuspension of RNA. Samples can be stored at −80°C prior to use in subsequent enzymatic reactions.

## Key Resources Table

**Table:** 

REAGENT or RESOURCE	SOURCE	IDENTIFIER
**Chemicals, Peptides, and Recombinant Proteins**		

Nuclease-free water (not DEPC-treated)	ThermoFisher	Cat#AM9932
NAD^+^	Roche	Cat#10127965001
NADH	Roche	Cat#10107735001
desphospho-CoA	Sigma-Aldrich	Cat#D3385
FAD	Sigma-Aldrich	Cat#F6625
3M Sodium Acetate, pH 5.5	ThermoFisher	Cat#AM9740
Glycogen from Oyster (type II)	Sigma-Aldrich	Cat#G8751
Ethyl Alcohol	Pharmco-AAPER	Cat#111000200
Isopropyl Alcohol	BDH	Cat#BDH1133-1LP
Low Range ssRNA Ladder	NEB	Cat#N0364S
SYBR Gold nucleic acid gel stain	ThermoFisher	Cat#S11494
Acid phenol:chloroform (CHCl_3_) pH 4.5	Fisher	Cat#BP17541
5X Detergent-free Phusion HF Buffer Pack	NEB	Cat#B0520S
RNaseOUT, Recombinant Ribonuclease Inhibitor	ThermoFisher	Cat#10777-019
Calf Intestine Alkaline Phosphatase	NEB	Cat#M0290S
RNA 5′ Polyphosphatase	Lucigen (Epicenter)	Cat#RP8092H
NudC	(Cahová et al., 2015)	N/A
Rai1	(Xiang et al., 2009)	N/A
T4 RNA Ligase 1 (ssRNA Ligase)	NEB	Cat#M0204L
Superscript III Reverse Transcriptase	ThermoFisher	Cat#18080-044
RNase H	ThermoFisher	Cat#AM2293
Phusion HF DNA Polymerase	ThermoFisher	Cat#F-530L

**Oligonucleotides**		

Illumina PCR primer, RP1: AATGATACGGCGACCACCGA GATCTACACGTTCAGAGTTCT ACAGTCCGA	Illumina	N/A
Illumina indexing PCR primer, RPI1 (index sequence is in bold): CAAG CAGAAGACGGCATACGAGAT**CGTGAT** GTGACTGGAGTTCCTTGGCA CCCGAGAATTCCA	Illumina	N/A
i105, 5′ adaptor with CUGA barcode and 11N extension (barcode is underlined): GUUCAGAGUUCUAC AGUCCGACGAUCCUGANNNNNNNNNNN	This paper	N/A
i106, 5′ adaptor with GACU barcode and 11N extension (barcode is underlined): GUUCAGAGUUCUACAGUCCGACGAUC GACUNNNNNNNNNNN	This paper	N/A
i107, 5′ adaptor with AGUC barcode and 11N extension (barcode is underlined): GUUCAGAGUUCUACAGUCCGACGAUC AGUCNNNNNNNNNNN	This paper	N/A
i108, 5′ adaptor with UCAG barcode and 11N extension (barcode sequence is underlined): GUUCAGAGUUCUACAGUC CGACGAUCUCAGNNNNNNNNNNN	This paper	N/A
RT primer: CCTTGGCACCCGAGAATTC CANNNNNNNNN	This paper	N/A
s1115, Custom Illumina Sequencing Primer: CTACACGTTCAGAGTTCTACAG TCCGACGATC	Illumina	N/A

**Other**		

Micellula DNA Emulsion PCR Kit	Chimerx	Cat#3600-02
Spin-X centrifuge tube filter, 0.45 μm, RNase/DNase free	Costar	Cat#8162
10% TBE-Urea gels, 1mm x 10 wells	ThermoFisher	Cat#EC6875Box
10% TBE gels, 1mm x 10 wells	ThermoFisher	Cat#EC6275Box

## Step-by-Step Method Details

***Note:*** The protocol for construction of cDNA libraries in CapZyme-seq enables single-nucleotide-resolution analysis of RNA 5′ ends. In particular, single-stranded oligonucleotide adapters are ligated to RNA 5′ ends and the 5′-adapter-ligated products are used as templates for generation of cDNAs that are analyzed by high-throughput sequencing. For each sequencing read, the precise identification of the RNA 5′ end sequence is accomplished by using the sequence of the 3′ end of the 5′ adapter as a point of reference.

### Enzymatic Treatments of RNA

**TIMING: ∼1-2 h**

CapZyme-seq relies on ligation of single-stranded oligonucleotide adapters to RNA 5′ ends prior to cDNA synthesis. This ligation step requires that the RNA have a 5′-monophosphate. Thus, analysis of RNAs without a 5′-monophosphate requires enzymatic processing of the RNAs to yield RNAs having a 5′-monophosphate. In CapZyme-seq the processing enzymes NudC and Rai1, which are specific for NCIN-capped RNA, along with RNA 5′ polyphosphatase (Rpp), a processing enzyme specific for uncapped, 5′-triphosphate RNA, are employed to enable differential detection and quantitation of NCIN-capped RNA and uncapped, 5′-triphosphate RNA.

**Figure 2:**
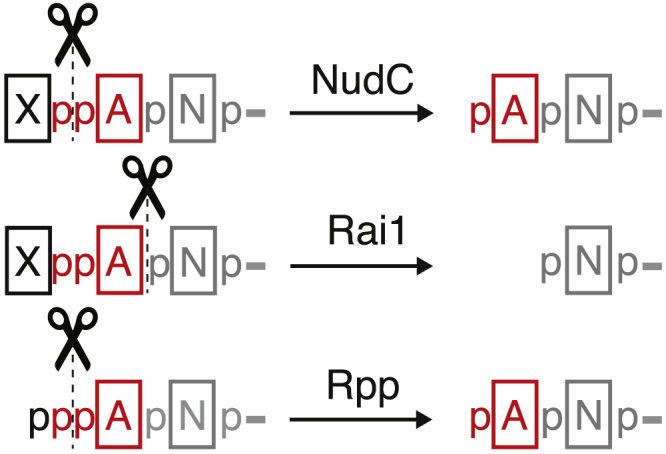
Enzymatic Processing of RNA 5′ Ends by Rpp, NudC, and Rai1

For selective processing of NCIN-capped RNA to 5′-monophosphate RNA, we use the bacterial NCIN-decapping enzyme NudC or the fungal RNA-decapping enzyme Rai1. NudC processes NCIN-capped RNA to 5′-monophosphate RNA by cleaving the diphosphate group of the NCIN cap, yielding products comprising 5′-pNp- (where N is the 3′ nucleoside moiety of the NCIN) followed by the remainder of the RNA (Cahová et al., 2015, Höfer et al., 2016). Rai1 processes NCIN-capped RNAs to 5′-monophosphate RNA by cleaving the phosphodiester bond connecting the NCIN cap to the remainder of the RNA, yielding products comprising 5′-p- followed by the remainder of the RNA (Jiao et al., 2017; Figure 2). NudC and Rai1 process RNA capped with at least four of the nucleoside-containing metabolites that can serve as NCINs: NAD^+^, NADH, dpCoA, and FAD (Bird et al., 2016, Cahová et al., 2015, Jiao et al., 2017, Vvedenskaya et al., 2018a).

a. Set heat block to 37°C.
b. Aliquot RNA into 1.7 mL nuclease-free tubes.

#### Rpp Treatment

For selective processing of uncapped, 5′-triphosphate RNA to 5′-monophosphate RNA we use the RNA processing enzyme RNA 5′ polyphosphatase (Rpp), which cleaves the phosphodiester bond between the triphosphate β and α phosphates, yielding products comprising 5′-p- followed by the remainder of the RNA (Figure 2). Reaction mixtures performed without addition of Rpp serve as a control.

a. Assemble the reactions as indicated below.

**Table:** 

Reagents	Rpp Treatment Volume (μL)	Mock Rpp Treatment Volume (μL)
RNA (generated *in vitro* or *in vivo*)	10	10
10x Rpp buffer	2	2
RNaseOUT, 40U/μL	1	1
Rpp, 20U/μL	1	-
H_2_O	6	7
**Total:**	20	20
b. Incubate at 37°C for 30 min.

#### NudC Treatment

NudC processes NCIN-capped RNA to 5′-monophosphate RNA by cleaving the diphosphate group of the NCIN cap, yielding products comprising 5′-pNp- (where N is the 3′-nucleoside moiety of the NCIN, for example the adenosine moiety of NAD^+^) followed by the remainder of the RNA (Figure 2). Reaction mixtures performed without addition of NudC serve as a control.

a. Assemble the reactions as indicated below.

**Table:** 

Reagents	NudC Treatment Volume (μL)	Mock NudC Treatment Volume (μL)
CIP-treated RNA (generated *in vitro* or *in vivo*)	10	10
10x NEB Buffer 2	2	2
RNaseOUT, 40U/μL	1	1
NudC, 55 μM	1.3	-
H_2_O	5.7	7
**Total:**	20	20
b. Incubate reactions at 37°C for 30 min.

#### Rai1 Treatment

Rai1 processes NCIN-capped RNAs to 5′-monophosphate RNA by cleaving the phosphodiester bond connecting the NCIN cap to the remainder of the RNA, yielding products comprising 5′-p- followed by the remainder of the RNA (Figure 2). Reaction mixtures performed without addition of Rai1 serve as a control.

c. Assemble the reactions as indicated below.

**Table:** 

Reagents	Rai1 Treatment Volume (μL)	Mock Rai1 Treatment Volume (μL)
CIP-treated RNA (generated *in vitro* or *in vivo*)	10	10
10x Rai1 buffer	2	2
100 mM MnCl_2_, prepared fresh	0.2	0.2
RNaseOUT, 40U/μL	1	1
Rai1, 3.4 μM	2	-
H_2_O	4.8	6.8
**Total:**	20	20
d. Incubate reactions at 37°C for 30 min.
  ***Note:*** As mentioned above, NudC and Rai1 process RNA capped with at least four of the nucleoside-containing metabolites that can serve as NCINs: NAD^+^, NADH, dpCoA, and FAD. Therefore, use of NudC or Rai1 enables detection of NAD^+^-, NADH-, dpCoA-, and FAD-capped RNA by CapZyme-seq.

### Recovery of RNA Products after Enzymatic Processing

**TIMING: ∼4 h**

#### RNA Products Generated *In Vitro*

a. Add 20 μL of 2x RNA loading dye to the sample and load along with a ssRNA ladder on a 10% TBE-urea slab gels. Run gel in 1x TBE buffer until bromophenol blue dye front reaches ∼1/2 of the length of the gel.
b. Incubate gel with SYBR Gold nucleic acid gel stain and visualize bands with UV transillumination. Excise RNA products of the expected size from the gel using a scalpel. Gels for distinct reactions should be stained in separate containers.
c. Elute RNAs from gel using the procedure described in (**A**, Isolation of RNA products, step 1-7).
d. Resuspend RNAs in 10 μL of nuclease-free water. Pipette the suspension and incubate on ice for at least 20 min to permit full resuspension of the RNA.
  ***Note:*** Considering the relatively low amount of RNA generated by *in vitro* transcription, we do not recommend acid phenol/chloroform purification after enzymatic treatments as this may result in significant RNA loss. Instead, we recommend gel purification as described above.
  **CRITICAL:** We do not recommend heating samples prior to gel loading as it may result in degradation of the 5′ end NCIN cap.
  ***Optional:*** If desired, GlycoBlue can be used to assist in the visualization of the RNA pellet. However, use of GlycoBlue interferes with quantification of cDNA by Qubit ssDNA and dsDNA Assay kits.

#### RNA Products Generated *In Vivo*

e. Add nuclease-free H_2_O to 200 μL, mix with 200 μL of acid phenol/chloroform pH 4.5 and purify RNAs using the procedure described in (**B**, RNA isolation, step 5a-f).
f. Resuspend RNA in 10 μL of nuclease-free water. Pipette the suspension and incubate on ice for 20 min to permit solubilization of RNA.

### 5′ Adaptor Ligation

**TIMING: ∼18-20 h**

**Figure 3:**
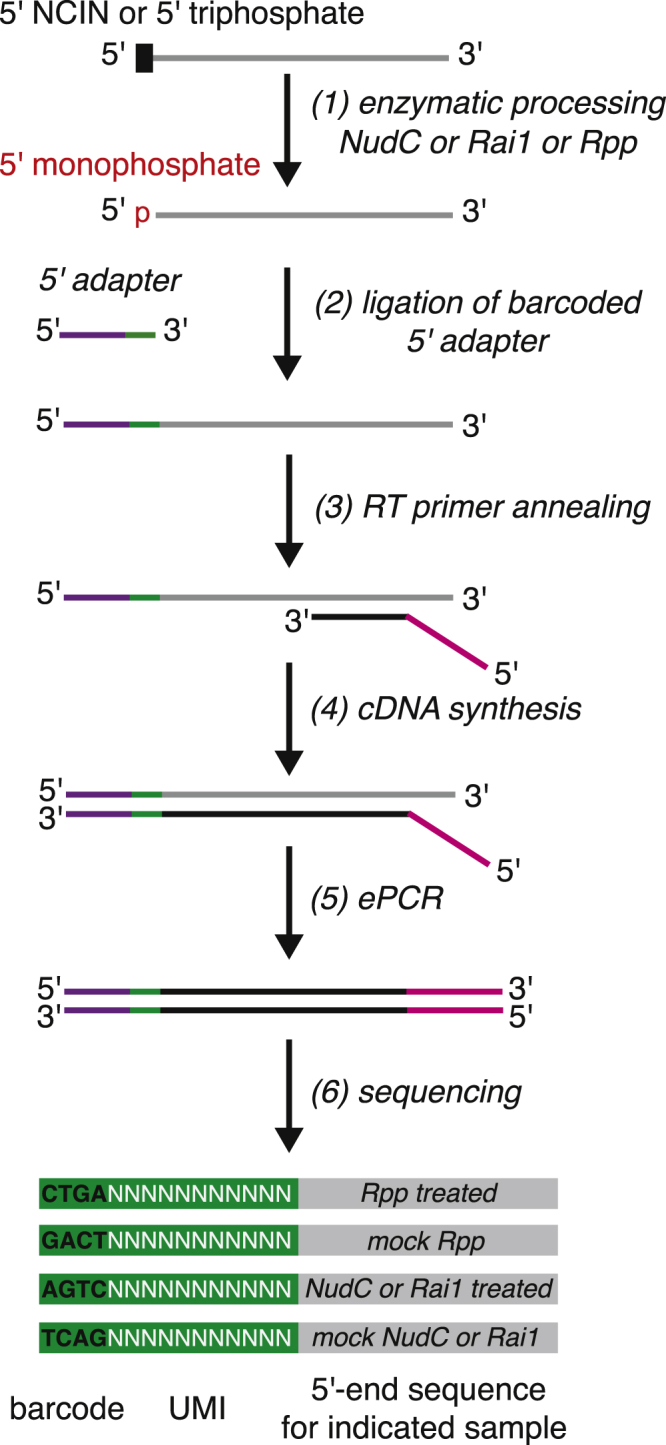
CapZyme-Seq Library Construction Workflow

CapZyme-seq^NudC^ involves quantitative comparisons between RNA samples treated with Rpp or NudC, along with two “mock” treated samples. CapZyme-seq^Rai1^ involves quantitative comparisons between RNA samples treated with Rpp or Rai1, along with two “mock” treated samples. Thus, to enable quantitative comparisons between the four samples in CapZyme-seq^NudC^ or CapZyme-seq^Rai1^, a unique, barcoded 5′-adaptor oligonucleotide is used in ligation reactions performed with each of the four individual samples (see Figure 3 and table below).

After the ligation reactions have been performed, the four samples of adaptor-ligated products are isolated by gel electrophoresis, combined together, and used for the remaining steps of library construction. After high-throughput sequencing, the identity of the 5′-adaptor barcode is used to associate each sequencing read with the sample from which it was derived.

The barcoded 5′-adaptor oligonucleotides also carry an extended randomized region that provides each sequencing read with a unique molecular identifier, “UMI,” enabling quantitative comparisons between cDNA libraries generated from RNA subjected to distinct enzymatic treatments. A typical CapZyme-seq experiment involves analysis of RNA subjected to four distinct treatments, and thus requires use of four barcoded 5′-adapters. Each barcoded 5′-adaptor contains a four nucleotide barcode followed by an 11-nucleotide randomized sequence. The barcode comprises the first four bases of each sequencing read while the 11-nucleotide randomized sequence comprises bases 5 to 15 of each sequencing read. Thus, the identity of the first four bases of each read is used to associate each sequencing read with a corresponding enzymatic processing reaction while bases 5 to 15 of each sequencing read are used as a UMI to reduce effects of PCR amplification bias.

**Table:** 

Sample	Barcoded 5′-adaptor oligonucleotide
Rpp-treated RNA	i105
mock Rpp-treated RNA	i106
NudC- or Rai1- treated RNA	i107
mock NudC- or Rai1-treated RNA	i108

1. Assemble ligation reaction as indicated below.

**Table:** 

Reagents	*in vitro* RNAs Volume (μL)	*in vivo* RNAs Volume (μL)
RNA (Rpp-, NudC-, Rai1-, or mock-treated)	10	10
5′ RNA adaptor (i105, i106, i107, or i108), 10 μM	1	3
10x T4 RNA ligase buffer	3	3
10 mM ATP	3	3
PEG8000, 50%	6	6
RNaseOUT, 40U/μL	1	1
T4 RNA Ligase 1, 10U/μL	1	1
H_2_O	5	3
**Total:**	30	30

***Note:*** The concentration of the 5′ adapter in reactions performed using RNA generated *in vivo* is three times greater than in reactions performed using RNA generated *in vitro*.

**Figure 4:**
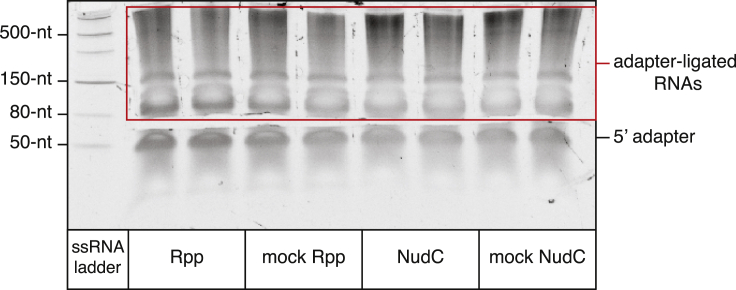
Representative Gel Stained with SYBR Gold Nucleic Acid Gel Stain
5. Excise 5′-adaptor-ligated products of the desired length from the gel using a scalpel.
6. Place gel slices into 4 to 8 tube-in-tube assemblies and recover nucleic acids using the procedure described in (**A**, Isolation of RNA products, step 3-8).
7. Resuspend adaptor-ligated products in nuclease-free water. (We recommend a volume of 15 μL for RNA generated *in vitro* and 30 μL for RNA generated *in vivo*). Pipette the suspension and incubate on ice for at least 20 min to enable full resuspension of the 5′-adaptor-ligated products.

***Note:*** For analysis of RNA products having a defined range of lengths, excise the portion of the gel slightly above and slightly below the desired size range. For analysis of all RNA products, excise the portion of the gel slighlty above the 5′ adapter to the top.

2. Incubate reactions at 16°C for 16 h.
3. Stop each reaction by adding 30 μL of 2x RNA loading dye. Load the four ligation reactions along with a ssRNA ladder on a 10% 7M urea slab gel. Run gel in 1x TBE buffer until the bromophenol blue dye front reaches ∼1/2 of the length of the gel.
4. Incubate gels with SYBR Gold nucleic acid gel stain and visualize bands with UV transillumination (Figure 4). Gels for individual ligation mixture should be stained in their own container.

### First-Strand cDNA Synthesis

**TIMING: ∼6-7 h**

Mix 15 μL of 5′-adaptor-ligated products with 0.3 μL of 100 μM of the reverse transcription (RT) primer (or a mixture of several RT primers to analyze several promoter sequences that are each upstream of a distinct transcribed region).

1. Incubate the mixture at 65°C for 5 min, then cool to 4°C.
2. Prepare the RT reaction mix as indicated below.

**Table:** 

Reagents	Volume (μL)
5x First-Strand buffer	6
10 mM dNTP mix	1.5
100 mM DTT	1.5
RNaseOUT, 40U/μL	1.5
SuperScript III Reverse Transcriptase, 200U/μL	1.5
H_2_O	2.7
**Total:**	14.7

3. Add mixture prepared in step 1 to mixture prepared in step 2. Pipette to mix. Incubate at 25°C for 5 min, 55°C for 60 min, 70°C for 15 min, then cool to 25°C.
4. Add 10U of RNase H and pipette to mix.
5. Incubate reaction at 37°C for 20 min then add 30 μL of 2x RNA loading dye.
6. Load the combined samples on a 10% 7M TBE-urea slab gel along with a ssRNA size ladder. Run gel in 1x TBE buffer until bromophenol blue dye front reaches ∼1/2 of the length of the gel. Incubate gels with SYBR Gold nucleic acid gel stain and visualize bands with UV transillumination. Gels for separate reactions should be stained in separate containers. Excise cDNAs of the desired length from the gel using a scalpel.
7. Place gel slices into 2 to 4 tube-in-tube assemblies and recover nucleic acids using the procedure described in (A, Isolation of RNA products, step 3–8).
8. Resuspend cDNAs in 20 μL of nuclease-free water. Pipette the suspension and incubate on ice for at least 20 min to enable the complete resuspension of cDNA. Measure the concentration of nucleic acids in each solution using Qubit ssDNA Assay kit (ThermoFisher).

**PAUSE POINT:** cDNA can be stored at −80°C for at least one week.

***Note:*** Ensure that any primers used for RT contain sequence complementary to the Illumina 3′ PCR primer.

### cDNA Amplification and Recovery

**TIMING: ∼7-8 h**

1. Dilute the cDNA with nuclease-free water to a concentration of ∼10^9^ molecules/μL.
2. Prepare emulsion PCR (ePCR) reaction mix as indicated below.

**Table:** 

Reagents	Volume (μL)
cDNA, ∼10^9^ molecules/μL	2
5x Detergent-free HF Phusion Buffer with MgCl_2_	10
0.1 mg/mL BSA	2.5
10 mM dNTP mix	2
10 μM Primer RP1	2.5
10 μM Primer RPI1-48	2.5
HF Phusion Polymerase, 2U/μL	1
H_2_O	27.5
**Total:**	50

3. Perform ePCR reactions using the recommended cycling conditions: 10 s at 90°C, 20 cycles of 5 s at 90°C, 5 s at 60°C, and 15 s at 72°C, followed by 5 min at 72°C.
4. Isolate cDNA from ePCR reactions using Micellula DNA Emulsion and Purification Kit. Elute cDNA from the column filter with 150 μL of the elution buffer provided with the kit. Add 15 μL of 3M NaOAc, 1.5 μL of glycogen (10 mg/mL) and 500 μL of 100% EtOH. Mix solution by vortexing and precipitate cDNA at −80°C for 16 h.
  **PAUSE POINT:** cDNA can be stored at −80°C for at least one week.
5. Centrifuge tubes at 4°C for 30 min to pellet cDNAs. Wash pellets 3 times with 1 mL of 80% ice-cold EtOH and resuspend cDNAs in 20 μL of nuclease-free water.
6. Mix 1 μL of the resuspended cDNA with 6x Orange DNA load dye and run a non-denaturing 10% TBE gel to check the library quality. Load O’Gene Ruler Ultra Low Range DNA ladder as size standard.
7. If residual primers are present, mix the rest of cDNA with 6x Orange DNA load dye and isolate amplified cDNAs by gel electrophoresis using a non-denaturing 10% TBE gel. Isolate products by gel excision using the procedure described in (**A**, Isolation of RNA products).
8. Elute cDNAs from gel with 600 μL of gel elution buffer at 37°C for 3 h, vortex the slurry every 30 min. Collect the eluate.
9. Add 60 μL of 3M NaOAc, 6.6 μL of glycogen (10 mg/mL), and an equal volume of isopropanol. Precipitate cDNAs at −80°C for 16 h.
10. Centrifuge samples at 4°C for 30 min to pellet cDNAs. Wash pellets 3 times with 1 mL of 80% ice-cold EtOH and resuspend cDNAs in 20 μL of nuclease-free water.
11. Measure the concentration of the amplified libraries using Qubit dsDNA HS Assay kit. Submit libraries for sequencing.

***Note:*** Use of ePCR minimizes the generation of amplicons derived from “template switching.”

***Note:*** Optimization of the amplification of cDNAs by ePCR is critical for successful library sequencing. In our experience, use of a Qubit ssDNA Assay kit to prepare ssDNA dilution of ∼10^9^ molecules/μl provides an imprecise measure of ssDNA concentration, likely because of chemicals in the solution that interfere with quantitation.

***Note:*** Barcoded libraries generated by CapZyme-seq are suitable for sequencing on Illumina NextSeq and Illumina HiSeq platforms in high-output mode. Sequencing primers provided by Illumina typically contain a mixture of several oligos. To avoid potential complications that may arise due to the presence of additional primers in the Illumina reagents we use a custom sequencing primer (s1115).

## Expected Outcomes

In published work, CapZyme-seq has been used to quantitate relative yields of NAD^+^-capped and uncapped RNA and to define preferred transcription start-site positions and consensus promoter sequences for NAD^+^ capping by bacterial RNAP *in vitro* and *in vivo* (Vvedenskaya et al., 2018a). The method can be readily adapted to define the promoter-sequence determinants for NCIN capping by bacterial RNAP holoenzymes carrying alternative σ factors, archaeal RNAP, eukaryotic RNAP I, II, and III, mitochondrial RNAP, and chloroplast RNAP.

The prevalence of nucleoside-containing metabolites that can function as NCINs (e.g., NAD^+^, NADH, dpCoA, FAD, UDP-glucose, and UDP-GlcNAc) underscores the need to determine, for each NCIN, the promoter-sequence determinants for NCIN capping. The CapZyme-seq method, either using the same decapping enzymes described here or using alternative decapping enzymes with alternative decapping specificities, should enable analysis of determination of NCIN-capping efficiencies and promoter sequence determinants thereof for each nucleoside-containing metabolite that can serve as an NCIN.

## Limitations

CapZyme-seq, in its current version that uses NudC and RaiI as decapping enzymes, identifies NCIN-capped RNA but does not identify the NCIN cap. This is not an issue for experiments *in vitro*, where the experimenter controls the identity of the NCIN. However, this can be an issue for experiments *in vivo*, where NCIN capping may occur with any of nucleoside-containing metabolites. Accordingly, for experiments *in vivo*, identification of the NCIN cap may require additional analysis (e.g., metabolic labeling, electrophoretic mobility, boronic-acid reactivity, immunoassay, or mass spectrometry [Bird et al., 2016, Cahová et al., 2015, Chen et al., 2009, Kowtoniuk et al., 2009, Nübel et al., 2017]). A goal of future work will be to develop alternate versions of CapZyme-seq that employ additional RNA 5′ end processing enzymes that selectively report on specific 5′ end modifications.

## Troubleshooting

### Problem

Multiple bands are visible on the gel below the 5′ adaptor during size selection of 5′-adaptor-ligated products.

### Potential Solution

Have adaptor resynthesized or perform gel purification of adaptor prior to ligation (load adaptor on a 10%-urea TBE slab gel, excise the major band from gel, elute and purify as described in A, Isolation of RNA products, steps 1–8).

### Problem

Insufficient amount of 5′-adaptor-ligated products.

### Potential Solution

Increase amount of starting material; collect all of the upper aqueous phase during the acid phenol/chloroform purification step.

### Problem

Insufficient yield of ePCR amplicons.

### Potential Solution

If the library after ePCR amplification is < 2.5 ng/μl as detected by Qubit dsDNA Assay kit, (i) perform two ePCR reactions per library; (ii) increase the volume of diluted ssDNA used in ePCR reaction by two-fold; (iii) increase the number of amplification cycles up to 30.

### Problem

A large portion of the library is comprised of a ∼150 bp product.

### Potential Solution

After 5′ adapter-ligated products or ssDNA have been subjected to gel electrophoresis, place the gel in SYBR Gold nucleic acid gel stain, incubate for 5 min with low agitation, and carefully excise products of the desired size. Alternatively, prior to incubation with SYBR Gold nucleic acid gel stain, remove the lower part of the gel containing an excess of 5′ adapters or an excess of RT primer(s), stain the remaining part of the gel, and carefully excise products of the desired size. For the final cDNA library, also perform gel electrophoresis, gel staining, and careful excision of products of the desired size.
